# Supplementary material for: Markov state models and NMR uncover an overlooked allosteric loop in p53
Source: Chem Sci. 2020 Dec 16;12(5):1891–900. doi: 10.1039/d0sc05053a (PMC8179107; doi:10.1039/d0sc05053a)
Supplement: SC-012-D0SC05053A-s001 [file SC-012-D0SC05053A-s001.pdf]

Electronic Supplementary Material (ESI) for Chemical Science.
This journal is © The Royal Society of Chemistry 2020

Supplementary Information

Markov State Models and NMR Uncover an Overlooked Allosteric Loop in p53

Emilia P. Barros,[a] Özlem Demir,[a] Jenaro Soto,[b] Melanie J. Cocco,[b,c] and Rommie E. Amaro*[a]

[a] Department of Chemistry and Biochemistry, University of California San Diego, La Jolla, CA, 92093, USA

[b] Department of Pharmaceutical Sciences, University of California Irvine, Irvine, CA, 92697, USA

[c] Department of Molecular Biology and Biochemistry, University of California Irvine, Irvine, 92697, CA, USA

* To whom correspondence should be addressed, email: ramaro@ucsd.edu

Materials and methods

System set up

The DNA binding domain initial coordinates were taken from chain B of PDB 1TSR, which include p53 amino acids 96 – 289. For the mutant simulations, the tyrosine in position 220 (125 in the clipped domain) was mutated to a cysteine using tleap module in Amber14.[1] The crystallographic water molecules were retained and each system was solvated in an 8Å TIP3P water box.[2] The zinc ion and its coordinating residues were modeled using the cationic dummy atom model.[3] Each system was brought to 0.12 M salt concentration by adding K^+ and Cl^- atoms. The structure file of each system consisted of about 27,220 atoms, which were prepared using Amber FF14SB force field.[1,4]

Molecular dynamics simulations

The solvated proteins were minimized and equilibrated as described in Malmstrom *et al,*[5] using the GPU version of Amber 14. To increase the conformational sampling, a round of accelerated MD simulations (aMD)[6] was performed from the equilibrated structure using Amber14. Each system was simulated for 100 ns and 10 structures were selected for each system by clustering the conformations based on RMSD of the center of mass of each residue using a k-means algorithm in MSMBuilder2[7] and using the cluster centroids. These 10 structures were used as seeds for short unbiased MD simulations, each performed in triplicate with new starting velocities. After each round of simulation, the joint trajectories were processed for MSM model construction, and new starting coordinates were selected, prioritizing the exploration of new areas in the conformational space, until converged models were obtained based on MSM validation metrics (see below). Individual simulations ranged from 10 to 300 ns in length. In total, the wildtype system was simulated for 89 μs, while Y220C required 63 μs for appropriate model construction.

Markov state model construction

Simulation data was processed and models were built using PyEMMA[8], version 3.5.6. Features consisted of pairwise distances, with pairs being selected after a tICA-based iterative process that eliminated redundant pairs located consistently close (< 3Å) or far (>10Å) in all frames of the simulations, as well as pairs involving residues located close to the clipped termini, with low variance (<0.05 Å) and those that accounted for low correlation with the first tICs (Supplementary Table 1). The final feature set consisted of 24 pairs (Supplementary Table 2). Time-independent component analysis (tICA)[9] was used to process the joint wildtype and Y220C featurized data. Distinct loop-centered Markov state models were constructed using the 17 features that are centered in L6 and the 7 for L1. Discretization was performed with k-means clustering, k = 200, for each system (wildtype and Y220C) separately, and accuracy of the models verified by implied timescale (ITS) plots and Chapman-Kolmogorov tests (Supplementary Figures 7 and 8). The L6

and L1-focused models were constructed with MSM lag time of 10 ns each. The MSMs were then coarse-grained using hidden Markov state models (HMMs) with a lag time of 3 ns (L1) or 2.5 ns (L6), again validated by ITS plots and Chapman-Kolmogorov tests (Supplementary Figures 9 and 10). The choice of macrostates for coarse graining of the MSMs was guided by the relative separation between neighboring timescales, favoring the number of macrostates that resulted in a timescale ratio above 1.5 with the next timescale. In cases where the consecutive timescales separation were not obvious, we verified the cluster assignments according to the different number of macrostates considered and the Chapman-Kolmogorov tests to decide on the final number of macrostates to proceed with. Standard deviations were calculated using Bayesian hidden Markov state models corresponding to the respective HMMs.

Pocket characterization

Pocket volume measurements were performed with POVME, version 2.0,[10] and druggability assessments were based on computational solvent mapping of randomly selected conformations from the MSM metastable states using FTMap.[11] Existence of hydrogen bonds across the simulations was probed using MDTraj,[12] with a hydrogen bond defined as established if donor-acceptor distance < 2.5 Å and angle > 120°.

MD ensemble comparison with experimental structures

For comparison of the conformations sampled by the simulations with experimentally-resolved p53 structures, we transformed the coordinates of wildtype and Y220C structures solved by X-ray crystallography and NMR spectroscopy applying the same feature and tICA transformation steps used for the L1 and L6 MSMs. A total of 58 wildtype and 54 Y220C chains were selected from 16 and 29 PDB entries, respectively, and are listed in Supplementary Tables 3 and 4. These structures were selected out of all wildtype and Y220C deposited structures as they contained the same number of Cα atoms to the simulation structures and thus could be directly compared in tICA space. The MD ensemble was also compared to the 2FEJ solution NMR structure's J-coupling and NOE-derived restraints. Distances corresponding to the 2,201 NOE pairs were calculated for the accrued wildtype simulations, and the fraction of frames that exceed the upper distance restraints were computed. Dihedral angles were calculated for the dihedrals identified by the J-coupling restraints, and violations computed when the average dihedral angle was outside the interval defined by the experimental value +- experimental error.

NMR data collection and sample preparation

WT p53 construct was provided by Rainer Brachman. ^{15}N-labeled p53 DBD was prepared similarly to Wong *et al.*[13] The DBD core domain of human p53 (94-312) was transformed into BL21 *E. Coli* cells. Bacteria were grown at 30 °C in ^{15}N-enriched Neidharts minimal media to a density of 0.8-1.0

$OD_{(550\ nm)}$. The temperature was lowered to 18 °C and both IPTG and ZnCl2 were added to a final concentration of 1 mM. Cells were allowed to grow for an additional 6-8 hours and then harvested by centrifugation. The frozen cell pellet was resuspended in 20mM sodium phosphate, pH7.2, 10 mM BME, 0.5 mM PMSF, and lysed using sonication. Cell debris were removed by centrifugation at 4 °C for 30 minutes. The supernatant was applied to a SP-sepharose column and the protein eluted with a gradient of 100 mM- 600mM NaCl. Samples were dialyzed into 15 mM potassium chloride, 25 mM sodium phosphate, pH 7.1, 10 mM BME and concentrated to a protein concentration of 400 μM.

All NMR experiments were performed on Varian Inova 800 MHz at 20 ^{o}C. NMR data were processed using nmrPipe.[14] Residues assignments and rate measurements (using peak volumes) in all 2D HSQC spectra were accomplished using CcpNmr Analysis.[15]

3D ^{15}N-TOCSY-HSQC (t_m = 75 ms) and NOESY-HSQC (t_m = 100 ms) were analyzed for assignment of backbone amide resonances. Published WT assignments[13] were confirmed in spectra of our WT sample.

Relaxation analysis

T1 and T2 spectra were recorded with relaxation delays of 10, 50, 100, 200, 400, 750, 1000, 1500 ms and 10, 30, 50, 70, 90, 110, 130, 150 ms, respectively. The NOE spectra were recorded with a 5 second irradiation and 3 second delay. R1 and R2 rates were obtain by measuring peak volume as a function of delay time and fitting them to a single exponential function with CcpNmr software.[15] Errors in relaxations rates were obtained through the covariance method in CcpNmr. R2/R1 error bars are a results of error propagation of both R1 and R2:

$$Error\ bar\ = R2/R1 \times \sqrt{\left(\frac{R1\ error}{R1}\right)^2 + \left(\frac{R2\ error}{R2}\right)^2}$$

NOE errors were calculated by the following equation:

$$Error\ = |NOE\ ratio| \times \sqrt{\left(\frac{NOE\ spectra\ noise}{NOE\ peak\ volume}\right)^2 + \left(\frac{reference\ spectra\ noise}{reference\ peak\ volume}\right)^2}$$

NMR generalized order parameters (S^2) were obtained by using *Modelfree 4.15*[16,17] in combination with *FASTModelfree.*[18] Initial estimation of *Modelfree* parameters were obtained through the programs *pdbinertia*, *r2r1_tm*, *quadric_diffusion* from the CoMD/NMR website.[19] Residues with NOE and X^2 values less than 0.6 and 10, respectively, were excluded from *quadric_diffusion* calculation. Our analysis was performed using the Protein Data Bank (PDB) file 2FEJ. All *FASTModelfree* analysis used an NH bond length of 1.015 Å and chemical shift entropy (CSA) value of -179 ppm similar to the previously published analysis of p53 DBD.[20] *FASTModelfree* analysis proceeded until all parameters converged.

Supplementary Table 1. Stepwise tICA-based selection of features for model building.

Iteration	Number of features	Number of tICs	Correlation cutoff	Constraints for next round
0	18,336	-	-	Remove pairs located < 3Å or > 10 Å apart in all frames
1	7,183	-	-	Remove pairs with distance variance < 0.05 Å
2	2,225	-	-	Remove pairs involving terminal residues
3	729	315	0.4	None applied
4	499	122	0.5	None applied
5	354	91	0.6	None applied
6	194	57	0.6	None applied
7	90	29	-	Remove features that involve residues close to termini
8	82	26	-	Remove similar pairs
9	35	16	0.75	Remove similar pairs
Final	24	13		

Supplementary Table 2. Pairs used for featurization of the simulations for model construction

Member 1 (Anchor residue)	Member 2
Ser116	Leu145
Ser116	Val147
Ser116	Thr150
Ser116	Tyr220
Ser116	Cys229
Ser116	Gly279
Ser116	Arg280
Pro223	Gly112
Pro223	Leu114
Pro223	Val143
Pro223	Leu145
Pro223	Thr230
Glu224	Pro153
Glu224	Gly154
Glu224	Cys229
Glu224	Ser260
Glu224	Ser261
Gly226	Thr155
Gly226	Arg156
Gly226	Pro219
Gly226	Tyr220
Gly226	Glu221
Gly226	Glu258
Gly226	Ser260

Supplementary Table 3. Wildtype X-ray structures used for comparison with simulations' conformational landscape

PDB ID	Chain ID

1GZH	C
1KZY	A,B
1TSR	A,B,C
1TUP	A,B,C
2AC0	A,B,C,D
2ADY	A,B
2AHI	A,B,D
2ATA	A,B,C,D
2H1L	M,N,O,P,Q,R,S,T,U,V,W,X
2OCJ	A,B,C,D
2XWR	A,B
2YBG	C,D
3KMD	A,B,C,D
3Q05	A,B,C,D
3TS8	A,B,C,D
4HJE	A,B,C,D

Supplementary Table 4. Y220C X-ray structures used for comparison with simulations' conformational landscape

PDB ID	Chain ID

2J1X	A
2VUK	A,B
2X0U	A
2X0V	A,B
2X0W	A
3ZME	A,B
4AGL	A,B
4AGM	A,B
4AGN	A,B
4AGO	A,B
4AGP	A,B
4AGQ	A,B
5A7B	A,B
5AB9	A,B
5ABA	A,B
5AOI	A,B
5AOJ	A,B
5AOK	A,B
5AOL	B
5AOM	A,B
5G4M	A,B
5G4N	A,B
5G4O	A,B
6GGA	A,B
6GGB	A,B
6GGC	A,B
6GGD	A,B
6GGE	A,B
6GGF	A,B

Supplementary Table 5. Persistence of L6-S3/S4 hydrogen bonds (in % of frames in the simulation)

Donor atom	Acceptor atom	Wildtype	Y220C
Thr149 - N	Gly225 - O	0	0.7
Thr149 - N	Asp227 – OD1	1.4	2.4
Thr149 - N	Asp227 – OD2	1.3	3.19
Cys219 – N	Thr154 - O	97.0	85.1
Ser226 – N	Thr149 – OG1	7.3	4.9
Asp227 - N	Thr149 – OG1	0.2	1.9
Thr149 – OG1*	Pro222 – O*	0.09	9.0
Thr149 – OG1	Val224 – O	0.02	1.0
Thr149 – OG1	Gly225 – O	0.04	1
Thr149 – OG1	Ser226 – OG	0.06	0.8
Thr149 – OG1	Ser226 – O	1.5	0.9
Thr149 – OG1	Asp227 – OD1	3.7	6.6
Thr149 – OG1	Asp227 – OD2	3.8	7.3
Thr149 – OG1	Asp227 – O	0.07	0.9
Thr154 – OG1	Cys219 - O	0.2	5.8
Ser226 - OG	Asp147 - O	0.01	1.1
Ser226 - OG	Thr54149 – OG1	0.3	1.0

* Interaction formed in the mutant-exclusive “sideways-bent” extended state

Supplementary Table 6. R1, R2, NOE, and S^2 values for Wildtype p53 DBD at 800 MHz field strength.

Residue #	R1 ± error (s^{-1})	R2 ± error (s^{-1})	NOE ± error	S2 ± error

99	1.485 ± 0.361	30.43 ± 0.654	0.795 ± 0.010	†
100	1.318 ± 0.284	28.36 ± 2.246	0.765 ± 0.012	0.791 ± 0.066
101	1.260 ± 0.275	31.81 ± 1.290	0.907 ± 0.006	†
102	1.209 ± 0.254	33.77 ± 4.850	0.657 ± 0.010	0.788 ± 0.089
103	1.087 ± 0.162	28.69 ± 0.811	0.711 ± 0.010	0.857 ± 0.027
104				
105	1.383 ± 0.287	35.68 ± 1.806	0.717 ± 0.009	0.645 ± 0.141
106				
107	1.255 ± 0.233	33.81 ± 2.730	0.820 ± 0.009	†
108	1.048 ± 0.213	42.69 ± 3.544	0.977 ± 0.010	†
109				
110	1.056 ± 0.206	31.40 ± 4.243	0.830 ± 0.013	1.000 ± 0.080
111	0.806 ± 0.119	33.32 ± 1.186	0.745 ± 0.010	0.905 ± 0.047
112	0.773 ± 0.184	**	1.000 ± 0.018	
113		28.78 ± 2.079		
114	0.979 ± 0.197	28.25 ± 1.185	0.775 ± 0.011	0.848 ± 0.036
115	1.718 ± 0.671	24.75 ± 3.405	0.379 ± 0.016	0.854 ± 0.091
116				
117	1.561 ± 0.461	30.35 ± 4.726	0.779 ± 0.018	†
118	1.990 ± 0.508		0.923 ± 0.016	
119				
120				
121	2.192 ± 0.573	29.47 ± 3.104	0.732 ± 0.014	†
122				
123	1.407 ± 0.434	34.21 ± 3.314	1.000 ± 0.027	
124	1.172 ± 0.201	29.86 ± 0.850	0.876 ± 0.009	1.000 ± 0.024
125	0.923 ± 0.148	28.53 ± 0.271	0.854 ± 0.009	1.000 ± 0.008
126	1.355 ± 0.274	26.99 ± 2.243	0.913 ± 0.012	1.000 ± 0.063
127	0.884 ± 0.240	26.60 ± 3.197	0.866 ± 0.015	1.000 ± 0.080
128				
129				
130	0.933 ± 0.192	76.46 ± 22.161	0.930 ± 0.011	†
131				
132	0.923 ± 0.160	34.34 ± 1.178	0.730 ± 0.009	0.858 ± 0.062
133	0.820 ± 0.205		0.775 ± 0.021	
134	0.892 ± 0.114	30.62 ± 3.896	0.817 ± 0.013	1.000 ± 0.060
135	1.061 ± 0.205	32.10 ± 2.578	0.784 ± 0.009	0.872 ± 0.071
136	0.953 ± 0.174	29.92 ± 5.053	0.764 ± 0.011	0.940 ± 0.078
137	0.860 ± 0.150	27.96 ± 1.204	0.743 ± 0.013	0.872 ± 0.038
138	0.690 ± 0.164	28.16 ± 3.482	0.885 ± 0.016	0.906 ± 0.089

139	1.054 ± 0.204	27.36 ± 1.265	0.820 ± 0.010	0.898 ± 0.033
140	0.996 ± 0.163	30.71 ± 1.633	0.785 ± 0.008	0.879 ± 0.042
141	0.925 ± 0.190	36.04 ± 2.876	0.907 ± 0.013	1.000 ± 0.054
142				
143	0.827 ± 0.069	29.74 ± 3.598	0.642 ± 0.010	0.848 ± 0.031
144	1.371 ± 0.230		0.541 ± 0.009	0.289 ± 0.279
145	0.692 ± 0.093	39.78 ± 3.833	0.855 ± 0.013	1.000 ± 0.060
146	0.878 ± 0.158	29.38 ± 1.939		
147	0.950 ± 0.098	30.32 ± 1.871	0.711 ± 0.008	1.000 ± 0.038
148	0.893 ± 0.153	28.80 ± 2.982	1.000 ± 0.014	†
149	0.865 ± 0.145	27.82 ± 1.366	0.918 ± 0.007	†
150	0.979 ± 0.167	28.69 ± 0.651	0.832 ± 0.006	0.932 ± 0.018
151				
152				
153				
154				
155	0.870 ± 0.124	32.19 ± 2.627	0.689 ± 0.007	0.934 ± 0.055
156	0.823 ± 0.106	31.88 ± 0.328	0.830 ± 0.010	0.937 ± 0.011
157	0.774 ± 0.078	30.59 ± 1.577	0.858 ± 0.011	0.979 ± 0.037
158	1.037 ± 0.201	36.18 ± 5.200	0.896 ± 0.011	1.000 ± 0.083
159	1.009 ± 0.270	33.48 ± 3.200	0.833 ± 0.013	1.000 ± 0.053
160	1.040 ± 0.178	33.32 ± 1.551	0.983 ± 0.014	†
161	0.759 ± 0.168	**	0.609 ± 0.016	
162	0.887 ± 0.173	**	0.864 ± 0.011	
163	1.114 ± 0.299	*	0.978 ± 0.023	
164				
165				
166				
167				
168		32.56 ± 0.841		
169	1.164 ± 0.257	38.47 ± 2.541	0.849 ± 0.010	1.000 ± 0.046
170	1.885 ± 0.445		0.645 ± 0.010	
171	1.265 ± 0.262	35.89 ± 2.385	0.836 ± 0.007	1.000 ± 0.054
172	1.123 ± 0.206	31.12 ± 2.398	0.835 ± 0.010	1.000 ± 0.051
173	1.220 ± 0.170	*	0.871 ± 0.019	
174	1.208 ± 0.277	37.97 ± 4.934	1.000 ± 0.011	†
175	1.256 ± 0.365	**	0.638 ± 0.016	
176				
177				
178		30.25 ± 0.508	1.000 ± 0.006	

179	0.845 ± 0.094	34.63 ± 4.812	0.871 ± 0.011	1.000 ± 0.061
180	1.100 ± 0.195	37.32 ± 0.588	0.855 ± 0.010	1.000 ± 0.014
181	0.984 ± 0.166	38.27 ± 1.342	0.888 ± 0.010	1.000 ± 0.022
182	1.325 ± 0.231	33.77 ± 0.584	0.826 ± 0.008	†
183				
184	1.862 ± 0.468		0.855 ± 0.011	
185	0.959 ± 0.191	30.68 ± 1.257	0.824 ± 0.008	0.938 ± 0.029
186	1.831 ± 0.361	25.83 ± 0.958	0.876 ± 0.009	0.776 ± 0.027
187	1.544 ± 0.275	27.05 ± 1.468	0.669 ± 0.008	0.540 ± 0.131
188	1.250 ± 0.223	25.80 ± 1.758	0.544 ± 0.007	0.641 ± 0.118
189	1.000 ± 0.172	25.45 ± 2.785	0.944 ± 0.017	†
190				
191				
192	1.266 ± 0.273	21.52 ± 2.605	0.776 ± 0.011	0.675 ± 0.071
193	1.133 ± 0.278	36.58 ± 1.683	0.837 ± 0.011	1.000 ± 0.028
194				
195	1.669 ± 0.747		0.815 ± 0.022	
196	0.821 ± 0.150		0.719 ± 0.013	
197				
198	0.830 ± 0.116	28.91 ± 1.925	0.725 ± 0.010	0.904 ± 0.042
199	1.069 ± 0.193	26.88 ± 0.249	0.782 ± 0.010	0.889 ± 0.010
200	1.137 ± 0.195	26.18 ± 0.654	0.779 ± 0.007	0.798 ± 0.069
201	1.060 ± 0.314	29.00 ± 1.992	0.807 ± 0.019	0.851 ± 0.053
202	0.799 ± 0.098	31.51 ± 1.085	0.836 ± 0.008	1.000 ± 0.021
203	0.844 ± 0.088	28.17 ± 1.721	0.653 ± 0.007	0.837 ± 0.033
204	0.839 ± 0.057	31.78 ± 1.478	0.795 ± 0.010	0.906 ± 0.024
205	0.755 ± 0.105	33.81 ± 1.180	0.890 ± 0.010	1.000 ± 0.021
206	0.968 ± 0.169	33.20 ± 3.624	0.656 ± 0.012	0.815 ± 0.072
207				
208	1.005 ± 0.186	20.76 ± 1.623	0.636 ± 0.008	0.761 ± 0.058
209	0.955 ± 0.213	30.69 ± 2.027		
210	1.116 ± 0.156	28.50 ± 1.283	0.692 ± 0.007	0.824 ± 0.072
211	0.870 ± 0.173	27.78 ± 1.765	0.756 ± 0.015	0.911 ± 0.044
212	0.986 ± 0.130	33.73 ± 4.092	0.807 ± 0.012	0.862 ± 0.042
213	1.062 ± 0.255	32.79 ± 0.978		
214	1.530 ± 0.371	27.03 ± 7.639	0.830 ± 0.017	1.000 ± 0.127
215	1.065 ± 0.218	*	0.802 ± 0.014	
216	1.323 ± 0.276	30.35 ± 1.261	0.761 ± 0.006	0.659 ± 0.088
217	0.824 ± 0.166	29.17 ± 5.349	1.000 ± 0.012	†
218	0.869 ± 0.111	35.61 ± 4.136	0.844 ± 0.011	1.000 ± 0.064

0.778 ± 0.090	25.48 ± 2.771	0.919 ± 0.010	†
0.694 ± 0.066	34.36 ± 2.672	0.766 ± 0.008	0.948 ± 0.025
0.982 ± 0.124	24.56 ± 0.762	0.482 ± 0.006	0.790 ± 0.026
1.173 ± 0.222	25.92 ± 0.488	0.303 ± 0.009	0.755 ± 0.017
1.476 ± 0.368	24.11 ± 2.166	0.528 ± 0.009	0.664 ± 0.065
1.143 ± 0.219	30.11 ± 1.104	0.805 ± 0.006	0.867 ± 0.025
1.375 ± 0.193	16.88 ± 0.410	0.727 ± 0.007	0.452 ± 0.017
0.909 ± 0.143	26.67 ± 0.665	0.891 ± 0.009	0.962 ± 0.022
0.773 ± 0.092	31.50 ± 1.835		
1.212 ± 0.167	13.47 ± 0.814	0.570 ± 0.007	0.360 ± 0.026
0.870 ± 0.164	26.40 ± 3.284	0.813 ± 0.013	0.909 ± 0.052
0.804 ± 0.132	32.41 ± 2.290	0.769 ± 0.010	0.933 ± 0.041
0.889 ± 0.224	27.46 ± 2.910	0.667 ± 0.015	0.889 ± 0.066
0.993 ± 0.222	**	0.789 ± 0.019	
0.922 ± 0.149	38.71 ± 2.767	0.828 ± 0.008	0.966 ± 0.037
0.910 ± 0.196	*	0.821 ± 0.020	
0.817 ± 0.265	30.28 ± 4.720	1.000 ± 0.020	†
1.390 ± 0.390	37.53 ± 1.806	0.818 ± 0.014	1.000 ± 0.053
1.290 ± 0.237	35.10 ± 1.120	0.857 ± 0.008	1.000 ± 0.028
	**	0.641 ± 0.009	
0.691 ± 0.349		0.441 ± 0.024	
1.356 ± 0.316	29.32 ± 4.274	0.752 ± 0.011	0.750 ± 0.098
1.238 ± 0.228	**	0.891 ± 0.017	
0.877 ± 0.152	32.91 ± 3.637	1.000 ± 0.016	†
0.777 ± 0.093	30.40 ± 3.035	0.794 ± 0.011	0.952 ± 0.041
0.905 ± 0.152	27.89 ± 6.521	0.749 ± 0.010	0.927 ± 0.060
0.735 ± 0.142	35.66 ± 3.189	0.804 ± 0.011	0.953 ± 0.045
1.019 ± 0.164	**	0.880 ± 0.011	
0.821 ± 0.151	32.81 ± 0.732	0.978 ± 0.014	†
0.797 ± 0.097	30.05 ± 4.498	0.744 ± 0.008	0.921 ± 0.038

1.313 ± 0.234	28.84 ± 1.114	0.708 ± 0.009	0.720 ± 0.116
1.147 ± 0.195	30.43 ± 0.760	0.679 ± 0.006	0.805 ± 0.096
1.112 ± 0.200	28.30 ± 1.457	0.684 ± 0.007	0.766 ± 0.091
0.873 ± 0.088	29.22 ± 1.271	0.763 ± 0.006	0.884 ± 0.031
0.935 ± 0.140	25.10 ± 1.018	0.836 ± 0.008	0.939 ± 0.024
0.870 ± 0.112	24.07 ± 2.226	0.679 ± 0.011	0.843 ± 0.041
0.767 ± 0.099	35.92 ± 4.275	0.798 ± 0.012	0.979 ± 0.050
1.042 ± 0.181	34.64 ± 3.027	0.706 ± 0.013	0.777 ± 0.079
0.755 ± 0.134	36.05 ± 0.948	0.777 ± 0.009	0.913 ± 0.051
0.949 ± 0.176	28.42 ± 1.107	0.839 ± 0.008	0.941 ± 0.024
0.887 ± 0.130	32.60 ± 2.098	0.834 ± 0.010	1.000 ± 0.036
0.876 ± 0.163	23.84 ± 2.207	0.990 ± 0.011	†
0.907 ± 0.154	33.35 ± 4.561	0.739 ± 0.012	0.948 ± 0.071
1.052 ± 0.308	**	0.748 ± 0.012	
0.917 ± 0.159	32.24 ± 3.800	0.831 ± 0.011	1.000 ± 0.068
1.334 ± 0.288	30.05 ± 1.155	0.700 ± 0.007	0.872 ± 0.038
1.037 ± 0.172	37.30 ± 4.563	0.704 ± 0.010	0.775 ± 0.079
0.969 ± 0.190	31.54 ± 2.781	0.885 ± 0.011	1.000 ± 0.054
1.014 ± 0.149	31.55 ± 1.412	0.893 ± 0.009	0.995 ± 0.028
0.907 ± 0.142	35.02 ± 4.009	1.000 ± 0.012	†
0.810 ± 0.133	32.90 ± 0.830	0.730 ± 0.007	0.892 ± 0.053
1.009 ± 0.151	30.11 ± 0.433	0.635 ± 0.005	0.899 ± 0.013
0.930 ± 0.124	29.24 ± 1.380	0.749 ± 0.007	0.879 ± 0.039
0.838 ± 0.105	32.58 ± 2.887	0.821 ± 0.009	0.909 ± 0.027
0.845 ± 0.107	28.96 ± 1.542	0.694 ± 0.007	0.904 ± 0.037
0.886 ± 0.101	32.04 ± 0.495	0.764 ± 0.006	0.853 ± 0.044
0.844 ± 0.066	27.71 ± 0.712	0.759 ± 0.006	0.868 ± 0.018
1.935 ± 0.323	18.56 ± 0.266	0.518 ± 0.004	
2.608 ± 0.507	16.17 ± 0.553	0.382 ± 0.005	
2.297 ± 0.365	12.48 ± 0.251	0.489 ± 0.002	
3.202 ± 0.661	9.95 ± 0.387	0.380 ± 0.003	

299	2.166 ± 0.255	6.84 ± 0.121	0.322 ± 0.002
300			
301			
302			
303	3.522 ± 0.807	9.57 ± 0.600	0.374 ± 0.009
304			
305	3.965 ± 0.757	10.16 ± 0.646	0.281 ± 0.005
306	4.271 ± 0.877	8.21 ± 0.434	0.286 ± 0.005
307	3.801 ± 0.700	8.44 ± 0.224	0.256 ± 0.004
308	2.345 ± 0.363	5.96 ± 0.151	0.012 ± 0.002
309			
310	3.730 ± 0.835	7.93 ± 0.599	
311		10.82 ± 0.652	0.291 ± 0.005

* Signal present up to .030 seconds
** Signal present up to .050 seconds
† *Modelfree* calculation did not assign a model to residue.
Blank spaces correspond to residues with relaxation values that could not be obtained.

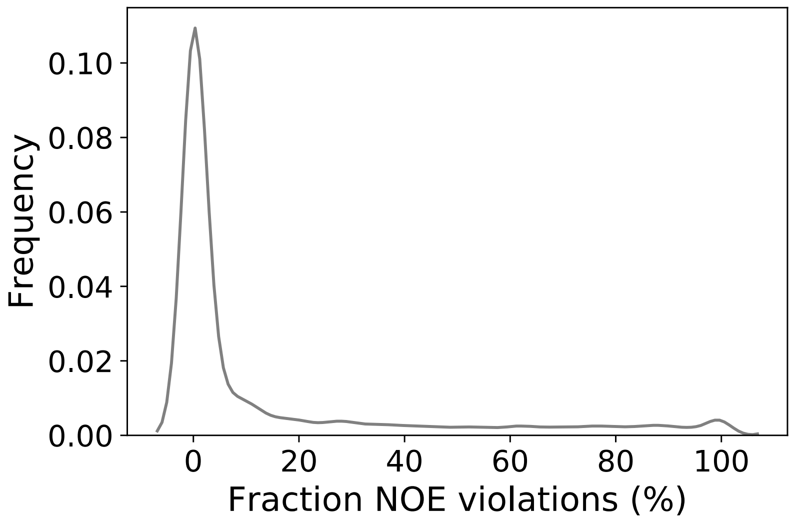

Supplementary Figure 1. Fraction of wildtype MD frames that violate the NOE upper distance restraint values for the 2,201 determined restraints from the NMR structure 2FEJ[21].

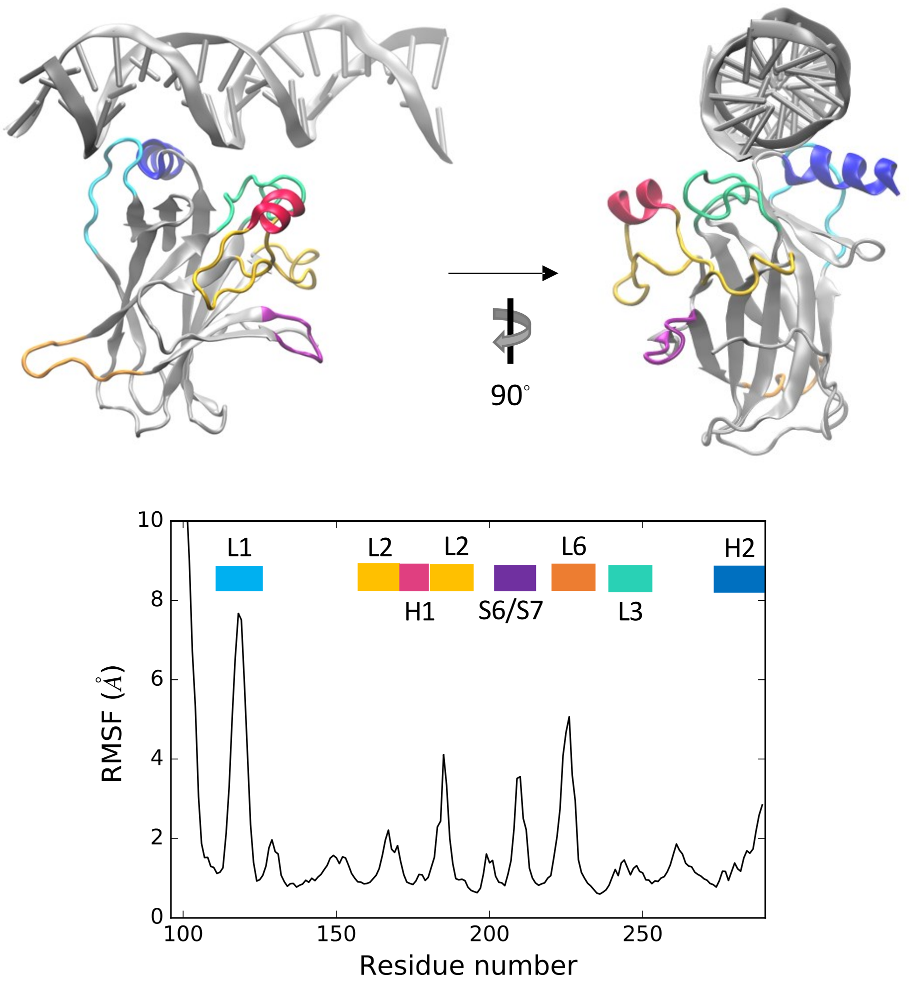

Supplementary Figure 2. Alpha carbon RMSF. Functionally important structural motifs are highlighted in the DNA-bound structure (top panel).

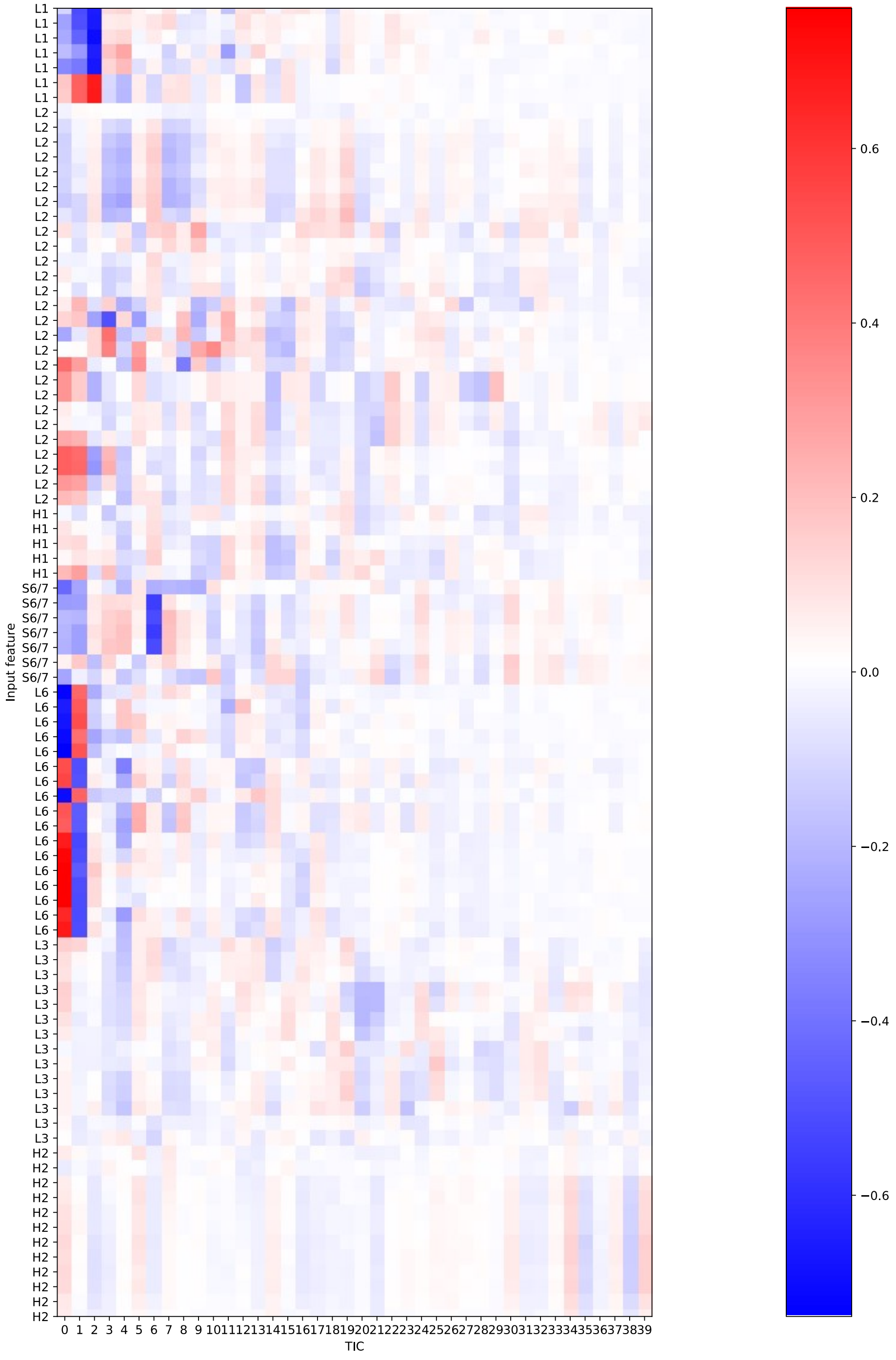

Supplementary Figure 3. tICA correlation for features incorporating functionally-important motifs in the protein (H1, H2, L2, L3, S6/7) in addition to L1 and L6.

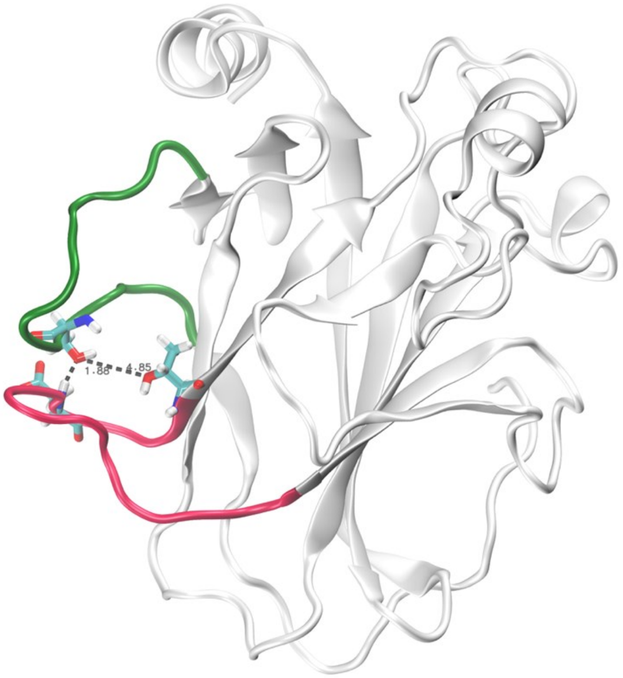

Supplementary Figure 4. Example of frame exhibiting most stable intra-loop hydrogen bonds, involving Ser116 in L1 and Asp228 or Thr231 in L6.

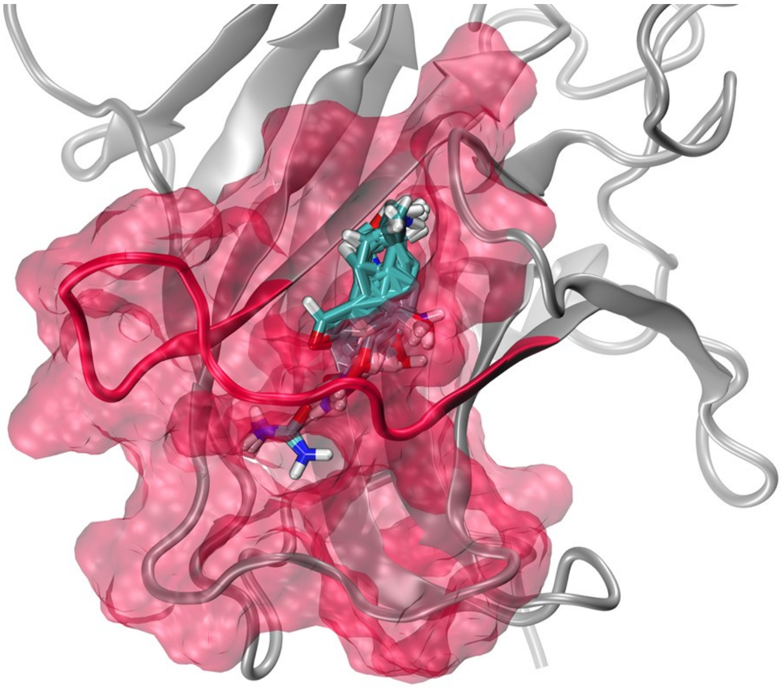

Supplementary Figure 5. Representation of the cryptic channel spanning loop L6 in the recessed Y220C metastable state. FTMap[11] probes indicating hotspots for drug binding are shown in licorice.

a b

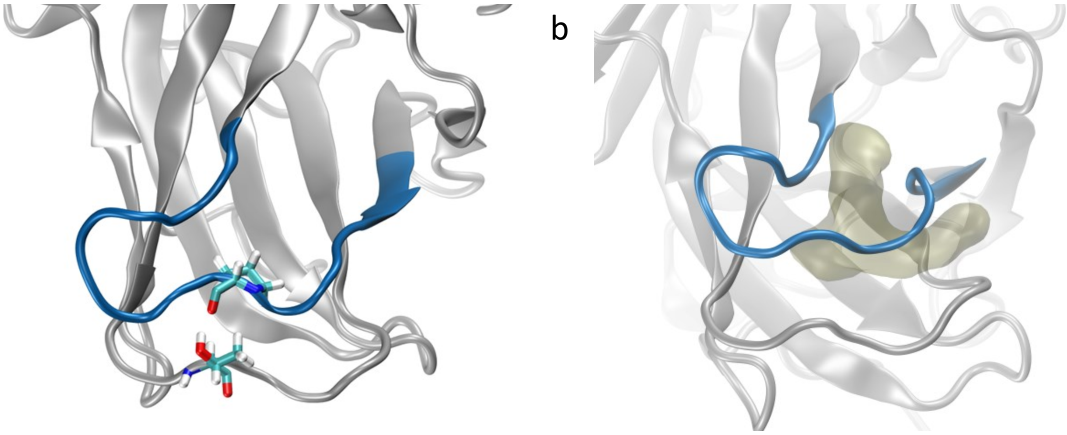

Supplementary Figure 6. (a) Representation of the Thr149-Pro222 interaction thought to stabilize the bent L6 conformation observed in the mutant-exclusive states. (b) Surface representation of the L6 pocket in the mutant-exclusive states.

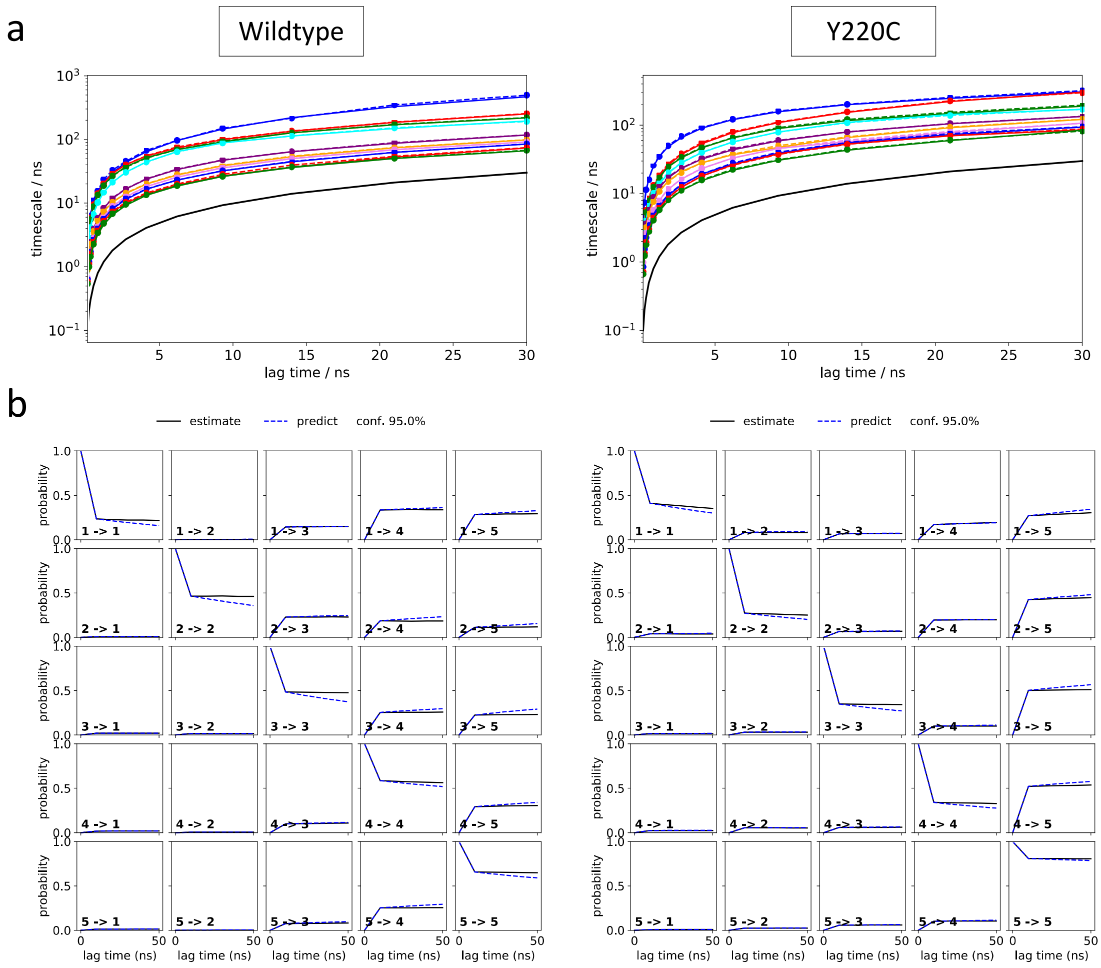

Supplementary Figure 7. L1 MSM model validation analysis: (a) Implied timescale plots and (b) Chapman-Kolmogorov tests.

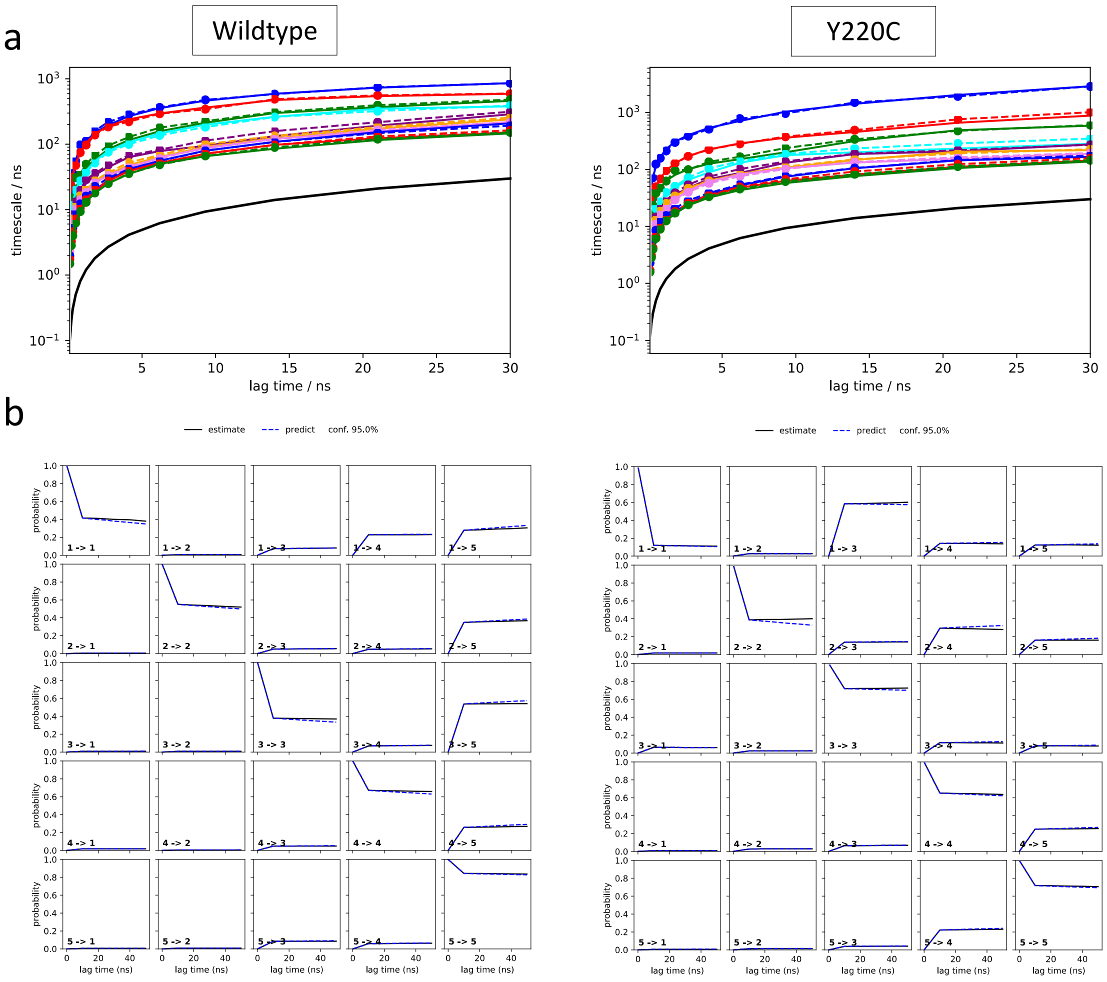

Supplementary Figure 8. L6 MSM model validation analysis: (a) Implied timescale plots and (b) Chapman-Kolmogorov tests.

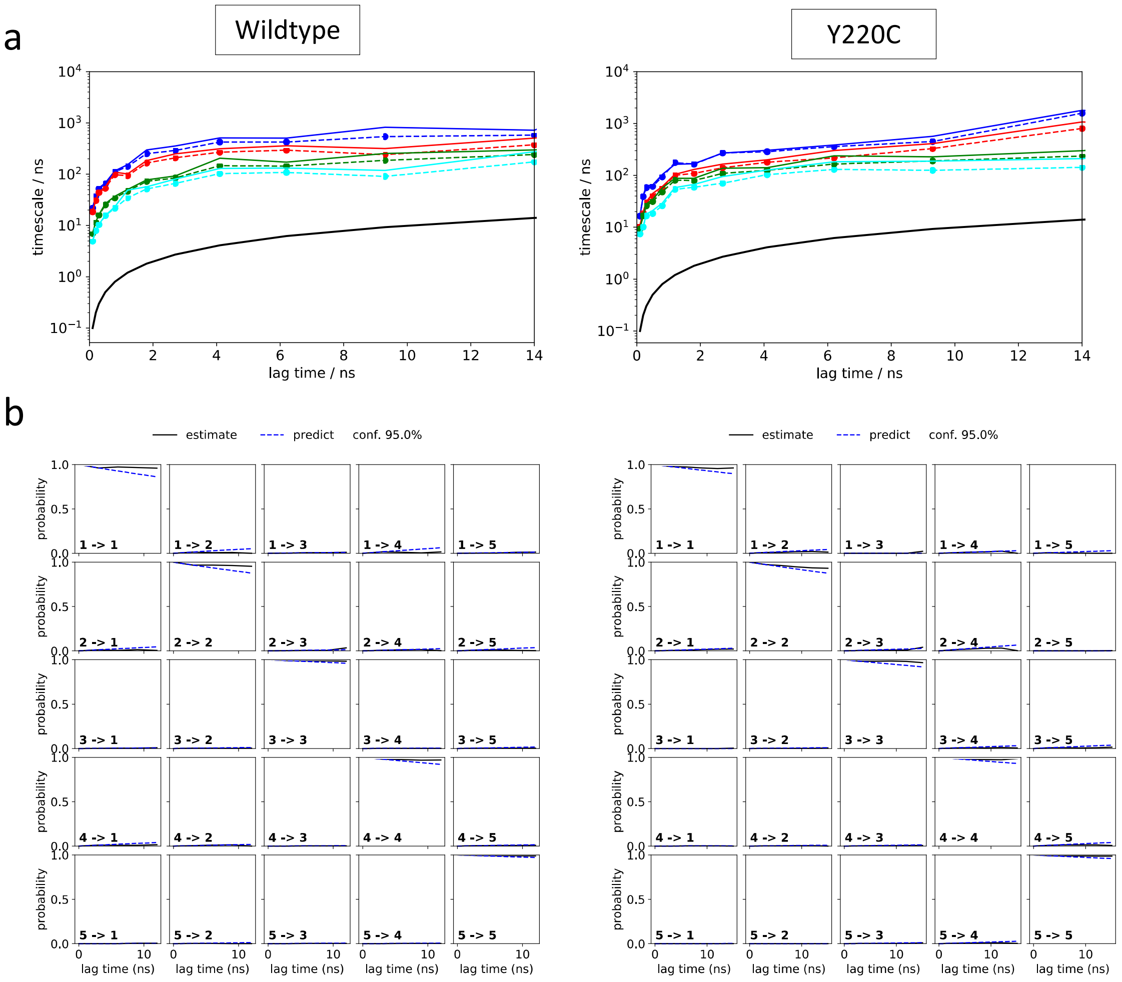

Supplementary Figure 9. L1 HMM model validation analysis: (a) Implied timescale plots and (b) Chapman-Kolmogorov tests.

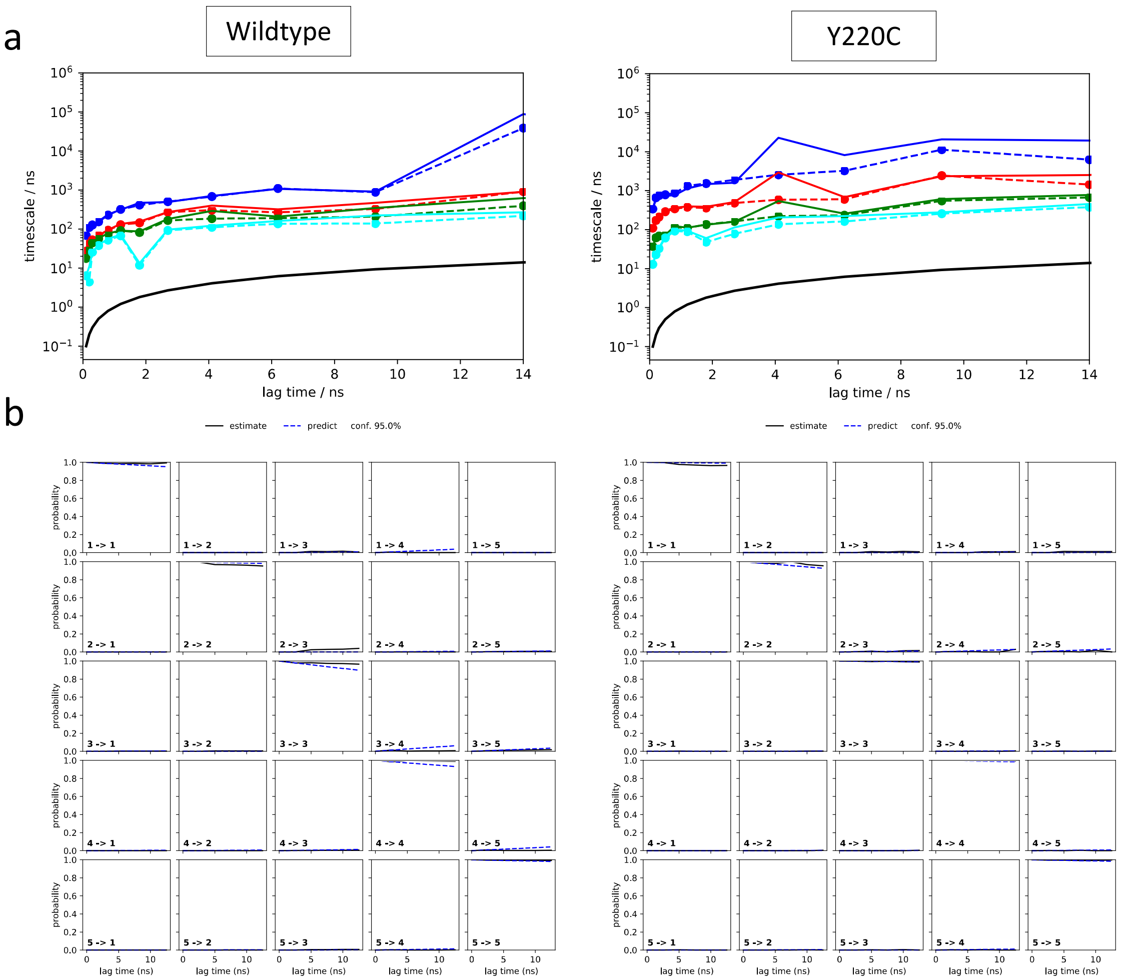

Supplementary Figure 10. L6 HMM model validation analysis: (a) Implied timescale plots and (b) Chapman-Kolmogorov tests.

References

1 D. A. Case, V. Babin, J. T. Berryman, R. M. Betz, Q. Cai, D. S. Cerutti, I. T.E. Cheatham, T. A. Darden, R. E. Duke, H. Gohlke, A. W. Goetz, S. Gusarov, N. Homeyer, P. Janowski, J. Kaus, I. Kolossváry, A. Kovalenko, T. S. Lee, S. LeGrand, T. Luchko, R. Luo, B. Madej, K. M. Merz, F. Paesani, D. R. Roe, A. Roitberg, R. C. Sagui, Salomon-Ferrer, G. Seabra, C. L. Simmerling, W. Smith, J. Swails, R. C. Walker, J. Wang, R. M. Wolf, X. Wu and P. A. Kollman, 2014.

2 W. L. Jorgensen, J. Chandrasekhar, J. D. Madura, R. W. Impey and M. L. Klein, *J. Chem. Phys*, 1983, **79**, 926–932.

3 Y. P. Pang, *J. Mol. Model.*, 1999, **5**, 196–202.

4 J. A. Maier, C. Martinez, K. Kasavajhala, L. Wickstrom, K. E. Hauser and C. Simmerling, *J. Chem. Theory Comput.*, 2015, **11**, 3696–3713.

5 R. D. Malmstrom, A. P. Kornev, S. S. Taylor and R. E. Amaro, *Nat. Commun.*, 2015, **6**, 7588.

6 L. C. T. Pierce, R. Salomon-Ferrer, C. Augusto F. De Oliveira, J. A. McCammon and R. C. Walker, *J. Chem. Theory Comput.*, 2012, **8**, 2997–3002.

7 K. A. Beauchamp, G. R. Bowman, T. J. Lane, L. Maibaum, I. S. Haque and V. S. Pande, *J. Chem. Theory Comput.*, 2011, **7**, 3412–3419.

8 M. K. Scherer, B. Trendelkamp-Schroer, F. Paul, G. Perez-Hernandez, M. Hoffmann, N. Plattner, C. Wehmeyer, J.-H. Prinz and F. Noe, *J. Chem. Theory Comput.*, 2015, **11**, 5525–5542.

9 G. Pérez-Hernández, F. Paul, T. Giorgino, G. De Fabritiis and F. Noé, *J. Chem. Phys*, 2013, **139**, 015102.

10 J. D. Durrant, L. Votapka and R. E. Amaro, *J. Chem. Theory Comput.*, 2014, **10**, 5047–5056.

11 D. Kozakov, L. E. Grove, D. R. Hall, T. Bohnuud, S. Mottarella, L. Luo, B. Xia, D. Beglov and S. Vajda, *Nat. Protoc*, 2015, **10**, 733–755.

12 R. T. McGibbon, K. A. Beauchamp, M. P. Harrigan, C. Klein, J. M. Swails, C. X. Hernandez, C. R. Schwantes, L.-P. Wang, T. J. Lane and V. S. Pande, *Biophys. J.*, 2015, **109**, 1528–1532.

13 K. B. Wong, B. S. DeDecker, S. M. V. Freund, M. R. Proctor, M. Bycroft and A. R. Fersht, *Proc. Natl. Acad. Sci. USA*, 1999, **96**, 8438–8442.

14 F. Delaglio, S. Grzesiek, G. W. Vuister, G. Zhu, J. Pfeifer and A. Bax, *J. Biomol. NMR*, 1995, **6**, 277–293.

15 W. F. Vranken, W. Boucher, T. J. Stevens, R. H. Fogh, A. Pajon, M. Llinas, E. L. Ulrich, J. L. Markley, J. Ionides and E. D. Laue, *Proteins*, 2005, **59**, 687–696.

16 A. M. Mandel, M. Akke and A. G. Palmer, *J. Mol. Biol.*, 1995, **246**, 144–163.

17 A. G. Palmer, M. Rance and P. E. Wright, *J. Am. Chem. Soc.*, 1991, **113**, 4371–4380.
18 R. Cole and J. P. Loria, *J. Biomol. NMR*, 2003, **26**, 203–213.
19 C. o. M. D. b. N. Spectroscopy, Tutorials at CoMD/NMR and NYSBC.
20 J. A. Rasquinha, A. Bej, S. Dutta and S. Mukherjee, *Biochemistry*, 2017, **56**, 4962–4971.
21 J. M. Pérez Cañadillas, H. Tidow, S. M. V. Freund, T. J. Rutherford, H. C. Ang and A. R. Fersht, *Proc. Natl. Acad. Sci. U. S. A.*, 2006, **103**, 2109–2114.
